# Survival of *Listeria* Strains and Shelf Life Determination of Fresh Blueberries (*Vaccinium corymbosum*) Treated with Cold Atmospheric Plasma

**DOI:** 10.3390/foods13060822

**Published:** 2024-03-07

**Authors:** Anibal A. Concha-Meyer, Alexandra González-Esparza, Patrick J. Cullen, Felipe Veloso, Mario Favre, Julio C. Valenzuela, Lorena Toloza, Brendan A. Niemira

**Affiliations:** 1 Instituto de Ciencia y Tecnología de los Alimentos, Facultad de Ciencias Agrarias y Alimentarias, Universidad Austral de Chile, Campus Isla Teja s/n, Valdivia 5090000, Chile; alexandra.gonzalez@uach.cl; 2 Centro de Estudios en Alimentos Procesados (CEAP), CONICYT-Regional, Gore Maule, R09I2001, Casilla 1007, Talca 3460000, Chile; 3 School of Chemical and Biomolecular Engineering, University of Sydney, Sydney 2006, Australia; patrick.cullen@sydney.edu.au; 4 Instituto de Física, Pontificia Universidad Católica de Chile, Casilla 306, Santiago 7820436, Chile; fveloso@uc.cl (F.V.); mfavre@fis.puc.cl (M.F.); jcvalenzuela@fis.uc.cl (J.C.V.); 5 Centro de Investigación en Nanotecnología y Materiales Avanzados CIEN-UC, Pontificia Universidad Católica de Chile, Santiago 7820436, Chile; 6 Department of Experimental and Health Sciences, Pompeu Fabra University, 08002 Barcelona, Spain; lorenaviviana.toloza@upf.edu; 7 USDA–ARS, Eastern Regional Research Center, Characterization and Interventions for Foodborne Pathogens Unit, Wyndmoor, PA 19038, USA; brendan.niemira@usda.gov

**Keywords:** *Listeria*, blueberries, cold atmospheric plasma, shelf life

## Abstract

Fresh blueberries are delicate, hand-picked, packaged, and refrigerated fruits vulnerable to spoilage and contamination. Cold atmospheric plasma (CAP) is a promising antimicrobial technology; therefore, this study evaluated the CAP treatment effect on acid-tolerant *Listeria innocua* and *Listeria monocytogenes* and evaluated changes in the quality of the treated fruit. Samples were spot-inoculated with pH 5.5 and 6.0 acid-adapted *Listeria* species. Samples were treated with gliding arc CAP for 15, 30, 45, and 60 s and evaluated after 0, 1, 4, 7, and 11 days of storage at 4 °C and 90% humidity for the following quality parameters: total aerobic counts, yeast and molds, texture, color, soluble solids, pH, and titratable acidity. CAP treatments of 30 s and over demonstrated significant reductions in pathogens under both the resistant strain and pH conditions. Sixty-second CAP achieved a 0.54 Log CFU g^−1^ reduction in *L. monocytogenes* (pH 5.5) and 0.28 Log CFU g^−1^ for *L. monocytogenes* (pH 6.0). Yeast and mold counts on day 0 showed statistically significant reductions after 30, 45, and 60 s CAP with an average 2.34 Log CFU g^−1^ reduction when compared to non-CAP treated samples. Quality parameters did not show major significant differences among CAP treatments during shelf life. CAP is an effective antimicrobial treatment that does not significantly affect fruit quality.

## 1. Introduction

Currently, Chile is an important berry producer in the southern hemisphere that has grown in blueberry exports, increasing from USD 30 million (around 4000 tons) in 2000 to USD 564 million (112,000 tons) in 2019, with destinations including the USA, China, South Korea, Australia, and numerous European countries [1,2]. Berries are delicate and can easily be spoiled due to mold and bacteria development by moisture. Therefore, it is not recommended to wash blueberries destined for the fresh market in order to maintain their good condition. Even small amounts of moisture can result in the rapid decay of the fruit, primarily due to the growth of grey mold (*Botrytis cinerea*) [3]. The foodborne bacteria *Listeria monocytogenes* can cause listeriosis, with symptoms including vomiting, diarrhea, and abortion during pregnancy [4,5]. Furthermore, the pathogen can be challenging to control in food since, as a facultative anaerobe, it can reproduce with or without oxygen, grow under refrigerated conditions, and develop resistance, such as an acid tolerance response. Due to outbreaks in the 1980s, authorities in the U.S. set a zero-tolerance requirement for ready-to-eat (RTE) foods contaminated with *L. monocytogenes* [6]. *Listeria* and RTE blueberries were linked to an outbreak of listeriosis in Connecticut in 1984 [7]. Moreover, a producer was forced to recall blueberries in California, Illinois, and Australia due to contamination with *L. monocytogenes* [8]. According to Desai et al. [9] blueberries are considered as novel food vehicles linked to *L. monocytogenes* transmission based on ProMED 1996–2018 reports. Although berries are reported to have antibacterial properties because of their acidity and phenolic compounds, based on extensive research with artificially contaminated berries, these properties should not be relied upon to ensure food safety [10,11,12]. Also, Chilean blueberries can take up to 20–50 days by sea freight to reach their final markets; therefore, postharvest measures are key to ensuring food safety and extending shelf life during storage and transportation [13,14,15].

Cold atmospheric plasma (CAP) is a non-thermal technology that has emerged as a potential method for microbial and chemical decontamination with successful results in controlling bacterial pathogens such as *Escherichia coli*, *Salmonella enterica* serovar Typhimurium, and *L. monocytogenes* on fresh vegetables and fruits [16,17]. However, the current literature lacks documentation of CAP effectiveness in controlling acid-adapted microorganisms on food products [18]. This postharvest treatment intervention offers the advantage of lacking chemical disinfection and being water-free, in addition to being able to operate openly and continuously at atmospheric pressure and temperature [19,20]. CAP has shown not to significantly affect the physicochemical properties of treated fruits and to extend the shelf life of berries up to 9 days at 4 °C [21].

Previous studies carried out by our research group have quantified the inhibition of *L. monocytogenes* and shelf-life extension of fresh blueberries under controlled atmosphere storage with a corona-discharge-generated ozone, which is one of the reactive oxygen species (ROS) produced by CAP [11,22]. However, the present study evaluated blowing arc CAP application since is a promising technology with high potential for berry sanitation considering the production of other antimicrobial agents including UV, reactive oxygen, and nitrogen species (RONS), electrically charged particles, and electric fields. Furthermore, CAP does not involve adding moisture or heat to the product, making it compatible with produce that is susceptible to changes in organoleptic properties. Moreover, this study objective was to evaluate CAP treatment effects on acid-tolerant *L. innocua* and *L. monocytogenes* and the shelf life of fresh blueberries and to perform an equipment characterization.

## 2. Materials and Methods

### 2.1. Cold Atmospheric Plasma Treatment Characterization

The CAP equipment characterization measurements were performed at the Institute of Physics, Pontificia Universidad Catolica de Chile, Santiago, Chile. A gliding arc plasma system operating with air as the feed gas including a fan to expel the gas at a flowrate of 10 LPM from the discharge zone (SAP 2, Surface LTDA, Campinas, Brazil) was used. This equipment was composed of two identical heads which operated individually. On each head, two cylindrical electrodes made of brass were placed parallel to each other with a separation of 18 mm, encapsulated together with the fan in a plastic container. These electrodes were connected to a power supply which provided an 8 kHz variable voltage amplitude of 5–10 kV. The equipment ran using a monophasic (domiciliary) 220 V, 50 Hz AC power line with a current consumption of 5 Ampers per operating head. Electrical parameters of the discharge were monitored using a high voltage probe (model PVM-5, North Star, Albuquerque, NM, USA) and a current probe (model TCP 0150, Tektronix, Beaverton, OR, USA) connected to a 500 MHz oscilloscope (model TDS 5054B, Tektronix, Beaverton, OR, USA) for full voltage waveform recording.

Additionally, equipment characterization included measuring optical emission spectroscopy (OES) using a UV-visible spectrometer (Ocean Optics USB2000, Dunedin, FL, USA) coupled to a UV-Vis optical fiber (Ocean Optics, Dunedin, FL, USA) to identify the electronic transitions in the gaseous species. Spectra recording of the light emission was performed in a line-of-sight parallel to the electrodes (~2 cm below them) along the discharge. Evaluation of the presence of different molecular species was performed by focusing the spectral measurements in the emission range between 200 nm and 800 nm. These results were complemented using the Specair software version 3.0.2.0 (Specair, Rhode-Saint-Genese, Belgium). This software generated a synthetic spectral curve using Boltzmann distributions for electronic, vibrational, and rotational transitions in the plasmas and/or reactive gases.

Finally, a thermal camera (model i5, Flir Systems Inc., Portland, OR, USA) with a 19 mm lens, a 60 Hz framerate, and with the measurable temperature in the range of 0 °C–250 °C was used to collect thermal data of the electrodes and the plasma.

### 2.2. Cold Atmospheric Plasma In Vitro Antimicrobial Assay

#### 2.2.1. Bacteria and Culture Conditions (Inocula Preparation)

Bacteria strains of *Listeria innocua* ISP 391-09 and *L. monocytogenes* PEEC 2015, provided by ISP Chile [23], were propagated in Brain Heart Infusion (BHI) broth (Oxoid, Hampshire, UK) and incubated overnight at 37 °C for propagation. After propagation, the cultures were centrifuged at 2680× *g* for 10 min at 4 °C (K241R, Centurion Scientific, West Sussex, UK) and the obtained pellet was resuspended in a 3 mL volume of BHI broths previously adjusted to pH 5.5 and 6.0 with lactic acid using a pHmeter (PHS-3BW, Bante Instruments, Shanghai, China) and incubated at 37 °C for 24 h. The later was performed following acid tolerance response induction methods used by Caggia et al. [24] and Concha-Meyer et al. [25] with modifications.

After incubation of *Listeria* species on pH-adjusted BHI broths, the cultures were centrifuged at 2680× *g* for 10 min at 4 °C to discard the supernatant and replace it with 2.5 mL of 0.1% peptone water. The solutions were resuspended using a vortex (Dragon Lab, MX-S, Beijing, China) and centrifuged again at 2680× *g* for 10 min at 4 °C. Afterward, the supernatant was discarded, and the pellet was dissolved in 2.5 mL of pH-adjusted BHI broths to a final concentration of 10^8^ CFU mL^−1^. Samples of different initial inocula were plated onto modified Oxford agar supplement (Difco, BD, Sparks, MD, USA) using an automatic spiral plater (Eddy Jet 2, IUL, Barcelona, Spain) to verify initial microbial counts.

#### 2.2.2. Fruit Sample Inoculation

Highbush blueberries (*Vaccinium corymbosum* L., cv. Legacy) were provided by Valle Maule S.A. (Parral, Maule, Chile) for in vitro antimicrobial assay. Fruits were visually selected by discarding damaged and immature fruits and choosing full-grown fruit in color (bluish). Subsequently, they were weighted in samples of 10 g (8 to 10 blueberries per sample). Prior to the inoculation of blueberries, they were oriented with their stem scar upwards inside a sterile petri dish. The elimination of the initial population of native microorganisms of blueberries was performed via exposure to UV light for 15 min inside a Class II Biological Safety Cabinet (BSC), type A2 (BSC-1500II A2-X, Biobase, Jinan, China). Each blueberry was inoculated on the surface of the stem scar using 15 µL of the initial inoculum whose concentration was approximately 10^8^ CFU mL^−1^ (BHI broth pH 5.5 and pH 6) with the respective *Listeria* species (*L. monocytogenes* and *L. innocua*) acid-adapted to pH 5.5 and 6.0. Blueberries were allowed to dry for 45 min in a BSC with laminar airflow at room temperature and relative humidity of 60%. Three biological replications were conducted, each consisting of fruit obtained on different dates.

#### 2.2.3. CAP In Vitro Antimicrobial Treatment

**Figure 1:**
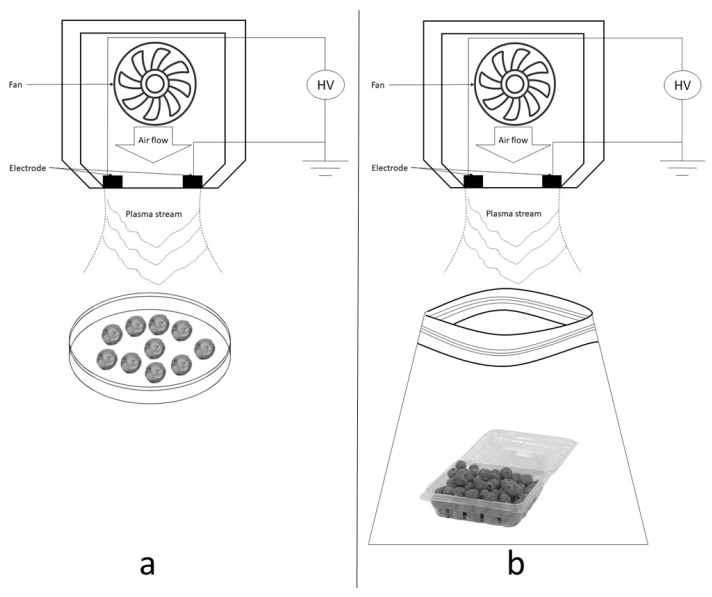
Scheme setup of CAP treatments of samples with blueberries: (**a**) CAP in vitro antimicrobial assay; (**b**) CAP shelf life extension assay.

Samples of inoculated blueberries were positioned stem scar upwards in a petri dish under the CAP system at a distance of 7 cm and treated for 15, 30, 45, and 60 s [26] (Figure 1a). Treatments were carried out in a BSC (BSC-1500II A2-X, Biobase, Jinan, China) at room temperature and relative humidity of 60%. As a control treatment, time 0 s equivalent to the initial bacterial count without CAP treatment was used.

#### 2.2.4. *L. monocytogenes* and *L. innocua* Recovery from Blueberries

The blueberries in each sample were transferred to a 16 oz sterile bag; 100 mL of 0.1% peptone water was added and then the sample was homogenized (Masticator, IUL, Barcelona, Spain) for 60 s. For the subsequent dilution of the obtained supernatant, 0.1% peptone water was used as the solvent and the 10^−1^ dilution was made, which was plated on oxford agar using an automatic spiral plater. Recounts of *L. monocytogenes* and *L. innocua* were determined after incubation of the plates for 24 h at 37 °C. Results were expressed in CFU g^−1^.

### 2.3. CAP Shelf Life Extension Assay

#### 2.3.1. Fruit Preparation

Highbush blueberries (*V. corymbosum* L., cv. Legacy) were provided by Hortifrut company for the shelf life study. Blueberries were weighed as 30 g samples and placed inside plastic clamshell containers, which were placed inside 27 × 28 cm resealable plastic bags (Ziploc, SC Johnson, Racine, WI, USA). Packaged samples were stored at a temperature of 5 °C in a refrigeration chamber.

#### 2.3.2. Cold Atmospheric Plasma Sample Treatment Conditions

The same gliding arc CAP equipment was used with the previously described operating conditions. Blueberries (30 g) in plastic clamshell container inside a resealable plastic bag were placed under the CAP equipment at a working distance of 7 cm from the base to the electrode (Figure 1b). With the clamshell container and overpack bag held open (see Figure 1), samples were treated with CAP for times of 0, 15, 30, 45, and 60 s, following previous experience with the equipment and methods followed by Lacombe et al. [17]. Immediately after CAP treatment, clamshell containers and bags were closed and stored at 4 °C and 90% relative humidity conditions (BJPX-HI 150ll, Biobase, Jinan, China) for 11 days.

#### 2.3.3. Microbiological Shelf Life Evaluation of CAP Treated Blueberries

CAP treated samples were evaluated for fruit shelf life after 0, 1, 4, 7, and 11 days of storage at 4 °C and 90% humidity. Samples were analyzed for total plate count of aerobic mesophilic organism and yeast and molds. Blueberries (10 g) were placed in sterile 20 × 30 cm stomacher bags with 100 mL of 0.1% peptone water and vigorously blended and homogenized in stomacher for 120 s (Masticator, IUL, Barcelona, Spain); then, samples were serially diluted and plated onto Tryptic Soy Agar (TSA) (Difco, BD Diagnostics, Sparks, MD, USA) and Potato Dextrose Agar (PDA) (Difco, BD Diagnostics, Sparks, MD, USA) using an automatic spiral plater (Eddy Jet 2, IUL, Barcelona, Spain). Plates were incubated for 24 h at 37 °C for TSA and 3 days at 25 °C for PDA. Results were expressed in Log CFU g^−1^.

#### 2.3.4. Quality Shelf Life Parameters Evaluation of CAP Treated Blueberries

The remaining 20 g of sample were used for quality controls. Texture analysis was carried out using a texture analyzer (TA.XTExpress, Stable Microsystems, Godalming, Surrey, UK) to measure puncture force with a 2 mm diameter stainless steel probe following procedures used by Concha-Meyer et al. [22]. Settings for the equipment were set to measure force in compression, with a probe height of 13 mm above the TA-90 base platform (Stable Microsystems, Godalming, Surrey, UK), transducer load of 50 kg, penetration of 3 mm, pre-test speed of 2 mm s^−1^, test speed of 1 mm s^−1^, automatic trigger force of 5 g, and post-test withdrawal speed of 2 mm s^−1^. The texture analyzer was force and height calibrated for every set of samples. Color measurements were made using a spectrophotometer (CM-5, Konica—Minolta, Osaka, Japan) and expressed using a CIE L*a*b* scale. Data were obtained in triplicate and interpreted using Spectramagic NX software version CM-S100W 2.03.0006 (Konica Minolta, Tokyo, Japan). Soluble solids content (°Brix) was determined using a digital refractometer (HI 96801, Hanna Instruments, Woonsocket, RI, USA). pH was determined using a calibrated pH meter (HI 8424, Hanna Instruments, Woonsocket, RI, USA). Titratable acidity was determined by homogenizing 3 g of fruit in 15 mL of deionized water and using titration with 0.1 N NaOH until pH 8.1 [27]. Results were expressed as a percent of total citric acid.

### 2.4. Statistical Analysis

All the analysis were performed in triplicate, and statistical analysis was conducted using statistical software (JMP 15, SAS Institute Inc., Cary, NC, USA). The randomized complete block factorial design with three replications was utilized to test the treatments and their interactions on *Listeria* counts, total plate count, yeast and mold counts, and texture firmness. Significance levels were defined as *p* ≤ 0.05, and variables were analyzed using analysis of variance (ANOVA) and mean separation via a Tukey HSD test. For the CAP in vitro antimicrobial assay, 18 samples were used for each *Listeria* species (9 samples for pH 5.5 and 9 samples for pH 6.0).

## 3. Results and Discussion

### 3.1. Cold Atmospheric Plasma Treatment Characterization

**Figure 2:**
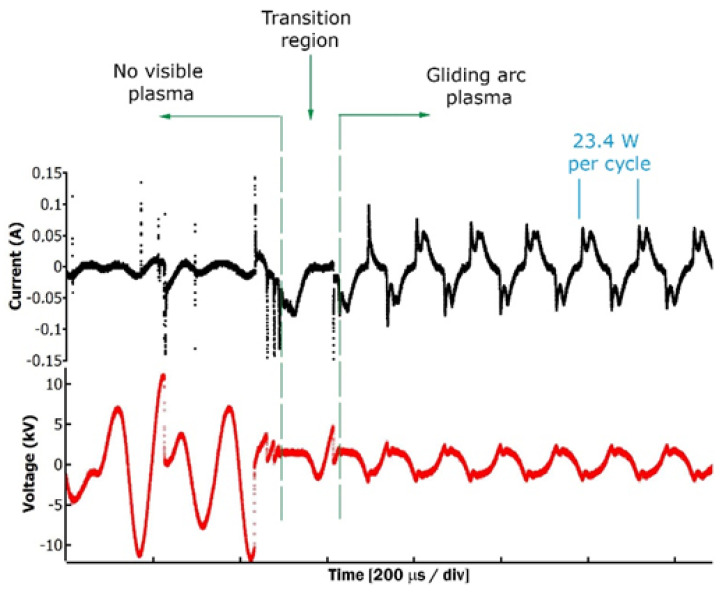
Current and voltage traces. Different regimes of operation, including no visible plasma discharge, transition region, and gliding arc plasma.

Once operating and prior to a visible plasma discharge appearing, both voltage and current showed variations in roughly random patterns (Figure 2). In this regime, sudden increases in current coincide with sudden voltage drops. Since the operating voltage of each head produces an electric field below the minimum required to induce electrical breakdown in air (E_breakdown_~30 kV cm^−1^), this observation indicates the random emission of electrons from the cathode without sufficient energy to produce ionization channels in the atmospheric gas [28]. This regime can be characterized as a dark discharge regime, where no visible conduction channels appear. Eventually, a transition region occurs on the operation of the power input, which is followed by a gliding arc plasma observable to the naked eye. The transition from a dark discharge regime to an operating gliding arc regime is enhanced by the augmented electron population and/or small aerosol particles pumped in the interelectrode region. In this regime of continuous plasma operation, which was employed in the following experiment conditions, the average power consumption for plasma production was 23.4 W per cycle.

**Figure 3:**
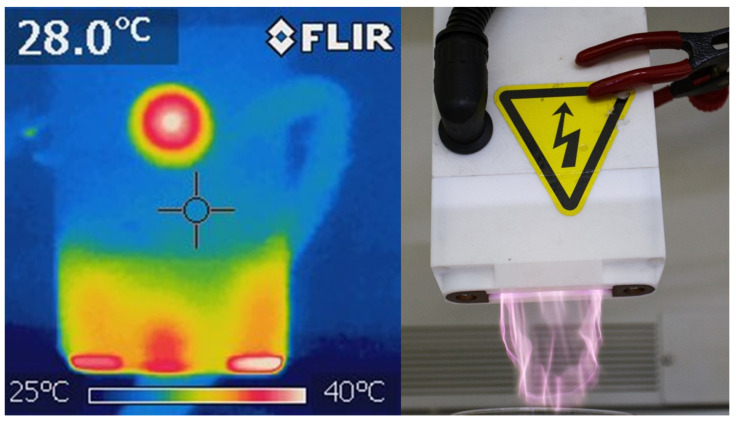
Comparison of thermal and non-thermal images of operating CAP electrode with visible plasma discharge.

Once operating, the gliding arc plasma can reach between 5 and 10 cm below the system housing, as shown in Figure 3. Furthermore, after 5 min of continuous operation, the temperature of the plastic housing stabilizes at 40 °C and no considerable temperature fluctuations are observed in the surrounding area (Figure 3).

**Figure 4:**
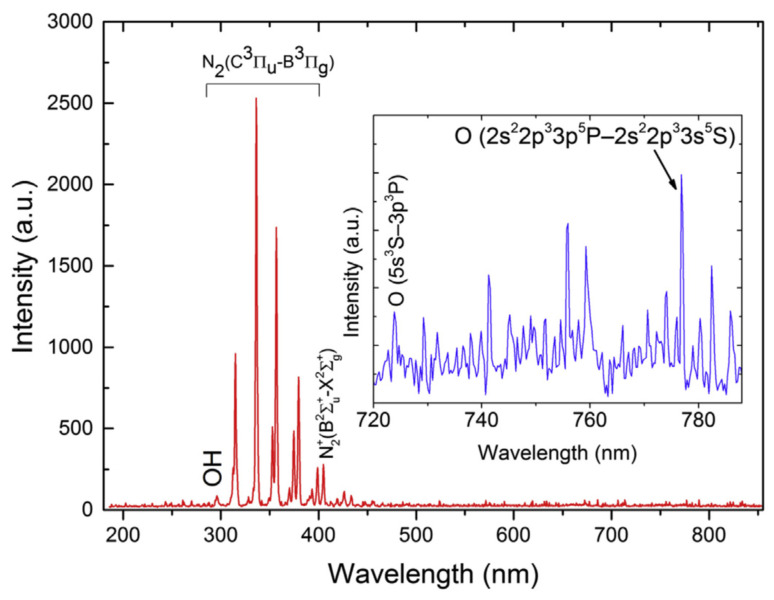
Optical emission spectrum measured from the gliding arc in the 200–850 nm range.

**Figure 5:**
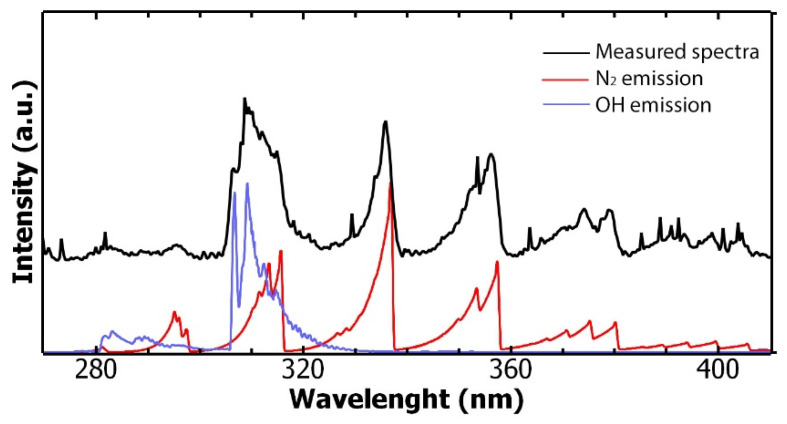
Measured spectral (black curve) of the gliding arc plasma, together with synthetic spectral curves of N_2_ (red curve) and OH (blue curve) using a molecular temperature of 5000 K.

Spectral measurements of the gliding arc discharge during operation are shown in Figure 4. A spectrum was obtained in the 200–850 nm range and showed a variety of emission lines. For example, the insert in Figure 4 shows emission lines from atomic oxygen, since plasma is hot enough to dissociate O_2_ molecules. Also, molecular bands in the 280–400 nm range are observed, which were further plotted for a better data analysis within this wavelength range (Figure 5). The spectrum includes a combination of two main emission bands, including hydroxyl radicals (OH-) with peaks at 307 nm and 309 nm and molecular nitrogen (N_2_). According to these spectra, molecular temperatures for OH- and N_2_ lie in the range 3000–5000 K with electron temperature T_e_ > 8000 K, whereas thermographic images (Figure 3) showed a maximum temperature of the equipment of 40 °C. These results indicate that gliding arc plasma is capable of producing high-temperature chemical reactions while maintaining relatively low temperatures in their surroundings due to the poor thermal conductivity of the plasma components [29].

### 3.2. Cold Atmospheric Plasma (CAP) In Vitro Antimicrobial Assay

CAP treatments over 30 s demonstrated significant reductions in both pathogens under both acid resistant pH conditions (Table 1). Log reductions of 0.54 CFU g^−1^ were achieved using 60 s CAP for *L. monocytogenes* ATR pH 5.5, while *L. monocytogenes* ATR pH 6.0 showed a reduction of 0.28 Log CFU g^−1^ after 60 s treatment. However, greater CAP treatment resulted in statistically significantly lower counts in both strains, and maximum count reductions were lower when compared to other authors. Niemira et al. [30] observed a 1.10 Log CFU g^−1^ reduction of *L. innocua* in golden delicious apples after a 4 min CAP treatment. Noriega et al. [20] reported CAP treatment for 8 min resulted in reductions for *L. innocua* of 1 log on chicken skin, and a 4 min treatment reached > 3 Log reductions on chicken muscle, which are significantly longer than treatment times used in this research. Ziuzina et al. [16] studied the inactivation of *L. monocytogenes* using CAP in cherry tomatoes, achieving an initial inoculum of 6.7 ± 0.6 log CFU sample^−1^, with similar initial counts when compared to this study. Further, a treatment for 45 s and 60 s reduced *L. monocytogenes* populations by 4.5 ± 0.2 Log CFU sample^−1^ and 5.1 ± 0.5 Log CFU sample^−1^, respectively. Finally, *L. monocytogenes* populations were reduced to levels below detection limits after prolonged treatment of 120 s, evidencing those reductions in bacterial populations followed a trend as treatment time was increased [16,31]. Nevertheless, Ziuzina et al. [31] and Ziuzina et al. [16] used a dielectric barrier discharge (DBD) system, while a gliding arc system was used in this study. Research suggests that the antimicrobial mechanism of CAP derives primarily from the production of UV and reactive chemical products such as reactive oxygen species (ROS) and reactive nitrogen species (RNS) [19]. According to Moldgy et al. [32] a gliding arc plasma provided enhanced antimicrobial properties when compared to DBD using equal power suggesting the low Henry’s law constant of NO as a reason, which is the predominant reactive species generated by a gliding arc. The authors claim that co-existence of O_3_ and NO_2_ in DBD treatments increased pathogen inactivation when compared to inactivation only by O_3_ or NO_2_ [32].

*L. innocua* initial counts showed an average of 0.53 Log CFU mL^−1^ lower values when compared to *L. monocytogenes* (Table 1), which could be associated with the absence of proteins regulated by glutamate decarboxylase (GAD) genes, which supports *Listeria* ability to grow at a low pH [33]. Also, for all CAP treatment times, both bacteria showed lower initial counts for ATR pH 5.5 when compared to ATR pH 6.0 (Table 1). Induced resistance to stress conditions, such as acid conditions, that would normally be lethal after short-term exposure could be beneficial when long-term sublethal stress conditions are applied. This may allow the activation of stress response regulators that control specific gene expression, which can trigger the production of cellular enzymes, synthesis of protective proteins and DNA against stress, and the modulation of the composition and physical properties of the bacteria [34]. Stress tolerance responses, including the ATR, represent a challenge to the food industry, especially when designing minimal processing regimes relying on the hurdles technology concept, where several stresses or hurdles are combined under mild conditions to control spoilage and pathogenic microorganisms [35]. The induction of these stress tolerance responses can result in cross-protection against a wide variety of lethal exposures other than the induction of the adaptive response. Such cross-protective responses are more relevant when exposure to sublethal stress conditions occurs in food processing, which can lead to the induction of multiple stress responses that can reduce the effectiveness of treatments [36]. This fact must be considered when implementing the application of novel conservation technologies such as CAP in ready-to-eat foods.

### 3.3. CAP Shelf Life Extension Assay

#### 3.3.1. Microbiological Shelf Life Evaluation of CAP Treated Blueberries

**Figure 6:**
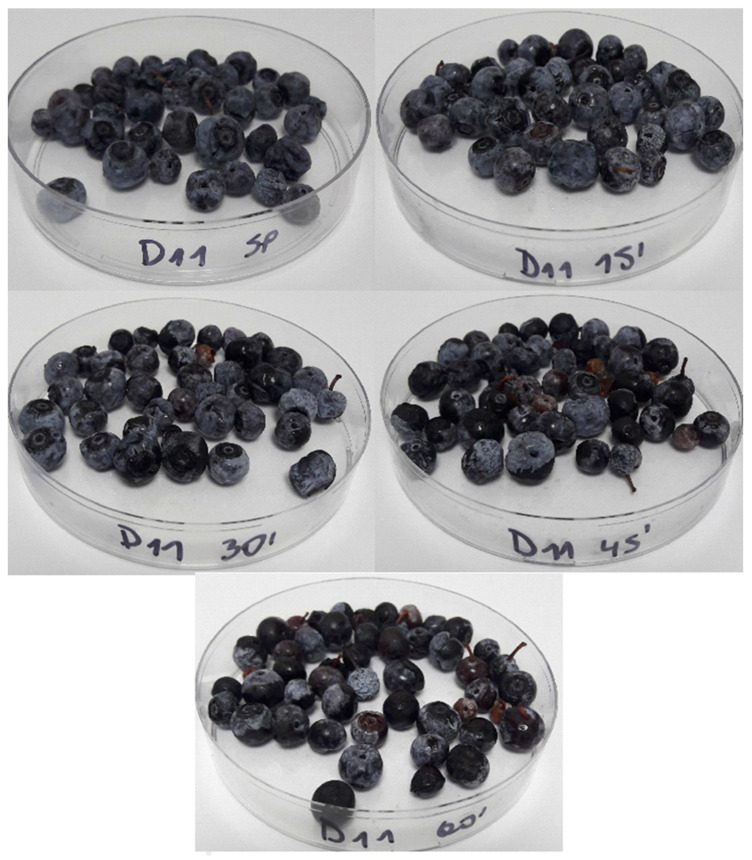
Visual appearance of samples treated with CAP and stored at 4 °C.

Yeast and mold (Y&M) counts (Table 2) on day 0 showed statistically significant reductions after 30, 45, and 60 s CAP treatments of samples when compared to non-CAP treated samples with an average 2.34 Log CFU g^−1^ reduction. However, on day 11, only Y&M counts of 30 s CAP treatment were significantly (*p* < 0.05) lower when compared to non-CAP treated samples with a 1.53 Log CFU g^−1^ reduction. Fluctuations in Y&M counts observed for 60 s CAP treatment can be attributed to low initial counts on blueberries, thus intensifying the visibility of variations even at a reduced baseline. Table 3 shows aerobic mesophilic bacteria counts, which presented very low native bacterial values (maximum growth of 1.40 ± 0.17 Log UFC g^−1^) and did not show statistically significant differences when comparing among treatments nor along storage days. Blueberries’ natural low pH values can serve as a hurdle for microbial growth; however, it does not impede yeast and mold growth [35]. Samples used in this experiment were obtained from factories that implement high food safety standards and comply with strict regulatory and commercial microbial quality policies; therefore, low native microbial counts could have interfered with estimating CAP treatment effect on aerobic mesophilic bacteria and Y&M. Pathak et al. [37] observed no statistically significant differences for Y&M when comparing untreated and 5 min treated blueberries after using a diffuse coplanar surface barrier discharge plasma treatment. Furthermore, CAP treatment reduced mesophilic aerobic bacteria largely in comparison to Y&M along a 10-day storage interval [37]. Lacombe et al. [17] used air-generated CAP treatment that achieved a statistically significant reduction in mesophilic aerobic bacterial values, while Y&M did not show significant reduction in blueberries, even for a 120 s CAP treatment duration during a seven-day storage period. Moreover, when comparing DBD-CAP reduction of total aerobic bacteria and Y&M, Dong and Yang [38] observed a weaker antimicrobial effect on Y&M that the authors linked to cell wall thickness and complexity of these microorganisms. Ji et al. [39] used DBD-CAP to treat blueberries and observed a significant decrease of 1.16 Log CFU g^−1^ Y&M after 90 s treatment, while for mesophilic aerobic bacteria a significant reduction of 0.88 Log CFU g^−1^ was obtained after the same treatment time. Mesophilic aerobic bacteria initial counts of blueberries in the latter study were 2.31 Log CFU g^−1^, which is higher than what was observed in our study [39]. However, Y&M CAP reduction values are comparable with our findings [39]. No visible microbial decay was observed in samples by day 11 (Figure 6), which correlates with low Y&M and aerobic mesophilic bacteria counts.

#### 3.3.2. Quality Shelf Life Parameters Evaluation of CAP Treated Blueberries

For instrumental texture (Table 4), no significant differences were observed in texture when comparing treatments within the same day of storage. However, all samples showed statistically significant differences when comparing day 0 and day 11, regardless of the treatment. These values demonstrate a tendency toward an increase in absolute strength to puncture the fruit by day 11, which is related to the loss of turgor and the difficulty of penetrating the waxy skin of blueberries due to fruit softening [40]. Other authors have reported a similar decrease in firmness when treating blueberries stored in containers using a plasma jet device [17] and DBD-CAP [40]. Firmness reduction is associated with softening of the fruit surface or damage to the outermost cells in the skin due to deterioration of internal structures, as well as potential mechanical injury or temperature damage during CAP treatment [17,41].

The color parameters resulting from the measurement in blueberries’ L*, a*, b*, ∆E, and C, indicating the level of luminosity, green/red coloration, blue/yellow coloration, color change, and saturation (bright or opaque), respectively, for the CAP treatment and shelf life storage times evaluated in this research (Table 5), show no significant differences. Therefore, quantitatively, the factors did not affect this fruit quality attribute. CAP 60 s treatment showed the highest values in color change for Day 4 and Day 11 (4.34 and 3.93). Pathak et al. [37] observed similar results when applying more extensive CAP treatments on blueberries using a diffuse coplanar surface barrier discharge (DCSBD) (up to 10 min) and concluded that there are no differences significant differences in the color parameters L*, a*, and b* with respect to the treatment times over the shelf life period studied. Hu et al. [42] worked with another equipment designed based on the surface discharge of the dielectric barrier (up to 20 min) and reported significant differences for treatment and storage times when evaluating shelf life. However, Lacombe et al. [17] applied CAP treatment times similar to this study from 0 to 120 s, using a device with two electrodes but with a customized version of a jet device, and placed the blueberry samples in a glass jar, thus concentrating the reactive species; they reported significant differences in the color parameters L*, a*, and b* for the CAP treatment times evaluated after its application. Likewise, Limnaios et al. [43] applied CAP for other berries such as red currants, using DCSBD equipment with treatment times of up to 10 min, and evaluated the useful life in a period of up to 10 days under these conditions. There were no significant differences in the color parameters L*, a*, and b* during the storage period for treatment times of 5 and 10 min, which was not the case for the samples without treatments where there were significant differences from day 1 to 10.

The differences reported in the literature for color may be attributed to different factors such as the type of equipment configuration, exposure times, storage time [37,42], the anthocyanin content of berries and the amount and structure of their wax [44,45]. There is a variability in the dependence relationship between color and anthocyanin content, where, in the case of cell rupture, there is an increase in the extraction of anthocyanins or a decrease when they are degraded via oxidative action and/or high temperatures [43].

The visual appearance of blueberries by day 11 (Figure 6) for 45 and 60 s treatments showed noticeable loss of bloom in some blueberries, in accordance with decreased L* and increased ∆*E* values (Table 5). Lacombe et al. [17] described an observable increase in reflectance of blueberries’ cutaneous wax after 45 s linked to treatment temperature that resulted in wax melting. Although L* values in our research showed a decrease over shelf life for 45 and 60 s treatments, changes were non-significant (*p* > 0.05). The bloom is a quality attribute of blueberries that provides a superficial appearance of a whitish waxy layer, and its preservation is an important objective during shelf life [46]. Therefore, CAP with direct application and longtime exposure can have a polishing effect over the surface, thereby removing bloom.

Titratable acidity of blueberries treated with CAP for 60 s showed a significant decrease (*p* < 0.05) when compared with non-treated blueberries on day 0 and 7; however, no significant changes (*p* > 0.05) were observed for the other days (Table 6). Other authors have observed no significant differences in the change for titratable acidity of control and CAP-treated fruit samples [40,47]. In this study, untreated samples showed a significant decrease (*p* < 0.05) in titratable acidity when comparing day 11 with the previous storage days (Table 6). According to Kader and Ben-Yehoshua (2000) the decrease in fruits’ titratable acidity during postharvest storage can be linked to the respiratory metabolism use of organic acids as substrates [48]. Nevertheless, values for 60 s CAP treatment samples on day 11 showed a significant increase (*p* < 0.05) when compared to day 0. Xiang et al. [49] observed an increase (*p* < 0.05) in titratable acidity in apple juice after a 90 W DBD plasma treatment for 200 s and related this phenomenon to a likely production of acidogenic molecules like nitric oxide.

Finally, CAP treatment showed no significant (*p* > 0.05) effect on total soluble solids and pH of blueberries during shelf life.

## 4. Conclusions

In this comprehensive research on CAP, the characteristics of the gliding arc equipment, including voltage, current, optical emission spectrum, and working temperature parameters, were determined. Additionally, the antimicrobial effect of CAP against *L. inoccua* and *L. monocytogenes* showed a statistically significant effect when samples were treated for 60 s. Listeria species tolerant to pH 5.5 showed greater susceptibility to CAP treatment. The shelf life of blueberries was not significantly affected by CAP treatment. Therefore, CAP emerges as a potent non-thermal antimicrobial treatment capable of inhibiting Listeria species without significantly compromising fruit quality. CAP has the potential to prevent pathogenic bacterial growth on fresh produce, especially on items like fresh blueberries that cannot be washed.

## Figures and Tables

**Table 1:** Mean count and standard deviation of *Listeria monocytogenes* and *Listeria innocua* with different pH ATR on inoculated fresh blueberries treated using different times of CAP.

	*Listeria innocua* (Log CFU mL^−1^)				*Listeria monocytogenes* (Log CFU mL^−1^)			
CAP Treatment (s)	ATR pH 5.5		ATR pH 6.0		ATR pH 5.5		ATR pH 6.0	
0	5.85 ± 0.02	A a	5.90 ± 0.02	B a	6.38 ± 0.01	C a	6.42 ± 0.02	D a
15	5.67 ± 0.03	A ab	5.86 ± 0.04	B a	6.30 ± 0.01	C a	6.40 ± 0.01	C ab
30	5.58 ± 0.04	A bc	5.67 ± 0.02	B b	6.15 ± 0.02	C b	6.22 ± 0.04	C bc
45	5.57 ± 0.02	A c	5.83 ± 0.01	B b	5.94 ± 0.02	C b	6.17 ± 0.01	C cd
60	5.45 ± 0.01	A c	5.57 ± 0.02	A c	5.84 ± 0.02	B c	6.14 ± 0.01	C d

Means within each row followed by the same capital letter are not significantly different (*p* > 0.05) from each other. Means within each column followed by the same lower-case letter are not significantly different (*p* > 0.05) from each other.

**Table 2:** Yeast and molds count (Log CFU g^−1^) in blueberries subjected to different times of treatments with CAP and stored at 4 °C.

CAP Treatment (s)	Day 0			Day 1			Day 4			Day 7			Day 11		
0	2.48 ± 0.20	A	a	2.85 ± 0.06	A	a	2.17 ± 0.03	A	a	2.25 ± 0.08	A	a	2.42 ± 0.07	A	a
15	2.76 ± 0.22	A	a	1.45 ± 0.15	B	b	<1.00 ± 0.00	B	b	1.30 ± 0.10	B	bc	3.01 ± 0.09	A	a
30	1.30 ± 0.10	A	b	2.60 ± 0.04	C	a	1.70 ± 0.17	ABC	a	1.30 ± 0.10	B	bc	1.07 ± 0.97	B	b
45	<1.00 ± 0.00	A	b	1.20 ± 0.10	A	b	<1.00 ± 0.00	A	b	<1.00 ± 0.00	A	c	2.00 ± 0.00	B	ab
60	<1.00 ± 0.00	A	b	2.33 ± 0.06	B	a	<1.00 ± 0.00	A	b	1.40 ± 0.17	B	ab	2.27 ± 0.03	B	ab

Means within each row followed by the same capital letter are not significantly different (*p* > 0.05) from each other. Means within each column followed by the same lower-case letter are not significantly different (*p* > 0.05) from each other.

**Table 3:** Aerobic mesophilic bacteria count (Log CFU g^−1^) in blueberries subjected to different times of treatments with CAP and stored at 4 °C.

CAP Treatment (s)	Day 0	Day 1	Day 4	Day 7	Day 11
0	<1.00 ± 0.00	1.30 ± 0.10	1.69 ± 0.09	<1.00 ± 0.00	1.30 ± 0.00
15	<1.00 ± 0.00	<1.00 ± 0.00	<1.00 ± 0.00	1.20 ± 0.10	1.40 ± 0.17
30	<1.00 ± 0.00	1.20 ± 0.10	1.60 ± 0.10	1.30 ± 0.10	1.30 ± 0.10
45	<1.00 ± 0.00	<1.00 ± 0.00	<1.00 ± 0.00	<1.00 ± 0.00	1.30 ± 0.10
60	1.45 ± 0.15	1.30 ± 0.10	<1.00 ± 0.00	1.30 ± 0.10	1.30 ± 0.10

Means within each row and columns did not show statistically significant differences (*p* > 0.05).

**Table 4:** Texture (Puncture Test Absolute Force g) of CAP treated blueberries stored at 4 °C.

CAP Treatment (s)	Day 0			Day 1			Day 4			Day 7			Day 11		
0	186.59 ± 65.54	A	a	177.55 ± 64.93	A	a	215.97 ± 61.37	AB	a	200.11 ± 79.92	A	a	296.57 ± 80.87	B	a
15	207.41 ± 56.19	AB	a	193.61 ± 47.60	A	a	268.77 ± 65.99	AB	a	264.88 ± 82.43	AB	a	285.45 ± 94.11	B	a
30	218.95 ± 56.83	A	a	185.56 ± 58.11	AB	a	231.42 ± 63.20	ABC	a	276.23 ± 65.02	BC	a	316.03 ± 61.10	C	a
45	179.60 ± 54.05	A	a	138.89 ± 40.57	A	a	200.85 ± 46.54	AB	a	279.62 ± 98.92	B	a	283.87 ± 84.11	B	a
60	158.04 ± 35.01	A	a	145.95 ± 46.24	A	a	203.73 ± 70.85	AB	a	271.72 ± 70.72	BC	a	315.59 ± 51.48	C	a

Means within each row followed by the same capital letter are not significantly different (*p* > 0.05) from each other. Means within each column followed by the same lowercase letter are not significantly different (*p* > 0.05) from each other.

**Table 5:** Color (CIE L*-a*-b*, ∆*E,* and *C*) parameters of CAP treated blueberries (*Vaccinium corymbosum*) stored at 4 °C.

Color Dimension	CAP Treatment (s)	Day 0	Day 1	Day 4	Day 7	Day 11
L*	0	43.70 ± 0.59	43.60 ± 0.10	43.54 ± 0.44	43.36 ± 0.51	42.42 ± 0.41
	15	43.70 ± 0.43	44.92 ± 0.32	42.94 ± 0.30	44.80 ± 0.58	42.56 ± 0.40
	30	44.77 ± 0.57	43.64 ± 0.16	43.27 ± 0.12	43.13 ± 0.27	40.81 ± 0.85
	45	44.07 ± 0.16	43.21 ± 0.45	42.58 ± 0.37	43.17 ± 0.67	40.03 ± 0.59
	60	43.30 ± 0.10	43.06 ± 0.16	39.61 ± 0.23	40.66 ± 0.49	38.79 ± 0.46
a*	0	0.04 ± 0.17	0.09 ± 0.23	0.82 ± 0.1	0.63 ± 0.07	0.2 ± 0.1
	15	0.23 ± 0.07	−0.09 ± 0.10	0.41 ± 0.12	0.27 ± 0.10	0.28 ± 0.12
	30	−0.17 ± 0.10	0.07 ± 0.10	0.21 ± 0.06	0.47 ± 0.19	0.40 ± 0.21
	45	−0.07 ± 0.03	−0.09 ± 0.17	0.19 ± 0.17	0.19 ± 0.15	0.76 ± 0.37
	60	0.43 ± 0.23	0.25 ± 0.03	1.15 ± 0.14	0.56 ± 0.22	0.89 ± 0.15
b*	0	−2.65 ± 0.19	−2.55 ± 0.20	−2.5 ± 0.44	−2.42 ± 0.1	−2.49 ± 0.04
	15	−2.23 ± 0.21	2.82 ± 0.04	−1.91 ± 0.26	−2.05 ± 0.08	−2.09 ± 0.24
	30	−2.39 ± 0.16	−2.40 ± 0.22	−1.66 ± 0.38	−1.56 ± 0.11	−1.94 ± 0.25
	45	−2.32 ± 0.06	−2.23 ± 0.11	−1.84 ± 0.17	−1.55 ± 0.17	−1.03 ± 0.50
	60	−2.14 ± 0.18	−1.75 ± 0.11	−0.79 ± 0.48	−0.76 ± 0.06	−1.16 ± 0.14
	0	0.00 ± 0.00	0.00 ± 0.00	0.00 ± 0.00	0.00 ± 0.00	0.00 ± 0.00
	15	0.68 ± 0.32	1.40 ± 0.11	0.96 ± 0.16	1.53 ± 0.34	0.45 ± 0.18
∆E*	30	1.38 ± 0.27	0.78 ± 0.70	0.93 ± 0.44	1.31 ± 0.61	1.82 ± 0.35
	45	0.72 ± 0.22	0.68 ± 0.25	1.34 ± 0.13	1.02 ± 0.05	2.89 ± 0.15
	60	0.84 ± 0.34	1.01 ± 0.27	4.34 ± 0.42	3.17 ± 0.04	3.93 ± 0.28
	0	2.65 ± 0.19	2.56 ± 0.20	2.64 ± 0.39	2.50 ± 0.08	2.50 ± 0.04
	15	2.24 ± 0.20	2.82 ± 0.04	1.96 ± 0.23	2.07 ± 0.06	2.11 ± 0.23
C*	30	2.40 ± 0.17	2.40 ± 0.22	1.68 ± 0.37	1.64 ± 0.04	1.99 ± 0.20
	45	2.32 ± 0.06	2.24 ± 0.11	1.86 ± 0.15	1.57 ± 0.15	1.37 ± 0.20
	60	2.19 ± 0.13	1.77 ± 0.11	1.44 ± 0.21	0.96 ± 0.09	1.47 ± 0.03

Means within each row and columns did not show statistically significant differences (*p* > 0.05).

**Table 6:** Titratable acidity (%) of CAP treated in blueberries (*Vaccinium corymbosum*) stored at 4 °C.

CAP Treatment (s)	Day 0			Day 1			Day 4			Day 7			Day 11		
0	0.58 ± 0.01	AB	a	0.64 ± 0.02	A	a	0.57 ± 0.01	AB	a	0.65 ± 0.06	A	a	0.48 ± 0.02	B	a
15	0.53 ± 0.02	A	ab	0.57 ± 0.04	A	a	0.59 ± 0.10	A	a	0.53 ± 0.03	A	abc	0.54 ± 0.02	A	a
30	0.47 ± 0.01	C	ab	0.67 ± 0.01	A	a	0.53 ± 0.01	B	a	0.59 ± 0.06	AB	ab	0.50 ± 0.09	BC	a
45	0.48 ± 0.02	B	ab	0.60 ± 0.05	A	a	0.60 ± 0.03	A	a	0.43 ± 0.02	B	c	0.51 ± 0.02	AB	a
60	0.43 ± 0.03	C	b	0.65 ± 0.03	A	a	0.63 ± 0.08	A	a	0.50 ± 0.03	BC	bc	0.59 ± 0.04	AB	a

Means within each row followed by the same capital letter are not significantly different (*p* > 0.05) from each other. Means within each column followed by the same lowercase letter are not significantly different (*p* > 0.05) from each other.

## Data Availability

The original contributions presented in the study are included in the article, further inquiries can be directed to the corresponding author.
